# Transmembrane protein 108 inhibits the proliferation and myelination of oligodendrocyte lineage cells in the corpus callosum

**DOI:** 10.1186/s13041-022-00918-7

**Published:** 2022-04-11

**Authors:** Yongqiang Wu, Yanzi Zhong, Xufeng Liao, Xiangguang Miao, Jianbo Yu, Xinsheng Lai, Yu Zhang, Chaolin Ma, Haili Pan, Shunqi Wang

**Affiliations:** 1 Institute of Life Science, Nanchang University, Nanchang, 330031 Jiangxi China; 2 School of Life Sciences, Nanchang University, Nanchang, 330031 Jiangxi China; 3 School of Basic Medical Sciences, Nanchang University, Nanchang, 330031 Jiangxi China; 4 Senior Middle School of Yongfeng, Ji’an, 343001 Jiangxi China; 5 Queen Mary School, Nanchang University, Nanchang, 330031 Jiangxi China; 6 Neurological Institute of Jiangxi Province and Department of Neurology, Jiangxi Provincial People’s Hospital, The First Affiliated Hospital of Nanchang Medical College, Nanchang, 330006 Jiangxi China

**Keywords:** *Tmem108*, Oligodendrocyte (OL), Myelination, Corpus callosum (CC), Bipolar disorder (BD)

## Abstract

**Background:**

Abnormal white matter is a common neurobiological change in bipolar disorder, and dysregulation of myelination in oligodendrocytes (OLs) is the cause. *Transmembrane protein 108* (*Tmem108*), as a susceptible gene of bipolar disorder, is expressed higher in OL lineage cells than any other lineage cells in the central nervous system. Moreover, *Tmem108* mutant mice exhibit mania-like behaviors, belonging to one of the signs of bipolar disorder. However, it is unknown whether *Tmem108* regulates the myelination of the OLs.

**Results:**

*Tmem108* expression in the corpus callosum decreased with the development, and OL progenitor cell proliferation and OL myelination were enhanced in the mutant mice. Moreover, the mutant mice exhibited mania-like behavior after acute restraint stress and were susceptible to drug-induced epilepsy.

**Conclusions:**

*Tmem108* inhibited OL progenitor cell proliferation and mitigated OL maturation in the corpus callosum, which may also provide a new role of *Tmem108* involving bipolar disorder pathogenesis.

**Supplementary Information:**

The online version contains supplementary material available at 10.1186/s13041-022-00918-7.

## Introduction

Bipolar disorder (BD) is a severe mental disease characterized by manic states being usually interspersed with periods of depression [1], affecting 1–1.5% of the population [1, 2]. Aberrant white matter microstructure is proposed as a mechanism underlying BD, including the dimension of irritability and widespread increases in radial diffusivity [3–5]. Abnormal white matter connectivity may be associated with BD pathophysiology [4, 6], and elevated rates of white matter hyperintensities are widely observed in BD [3–5].

The corpus callosum (CC) is the brain's major white matter fiber tract [6, 7], containing most axonal transmissions between the two cerebral hemispheres. The CC is among the last brain structures to complete myelination [8], which is also a period accompanying the peak onset of BD [9]. Anatomical abnormalities in the CC have been reported in magnetic resonance imaging studies in BD patients, possibly because of altered myelination leading to impaired interhemispheric communication [10]. Changes in area and thickness in the CC have been reported in BD, and neuropathological data and imaging suggest possible abnormalities in myelination and glial function [2, 9]. Given the strong genetic underpinnings of both BD and white matter microstructure, such white matter aberrations may be a disease marker and an endophenotype of BD [3, 11].

Several genome-wide association studies (GWAS) suggest that *Tmem108* is a susceptible gene of BD [12–14], and the relevant single nucleotide polymorphism (SNP) site is not in the coding region of *Tmem108*, which is speculated that the SNP may affect its expression [14, 15]. Strikingly, the recent GWAS screened BD risk loci in the Han Chinese population, covering 1822 BD patients and 4650 control individuals, and the data was replicated analysis. After finally multiple analyses between Han Chinese and European populations, a new SNP (rs9863544) in BD patients were found, locating in the upstream regulatory region of the *Tmem108* gene [14]. *Tmem108* expression change may be one of the onset reasons for BD.

Our previous research found that adult neurogenesis is impaired in *Tmem108* null mice, and manic behavior is found in the mutant mice [16], indicating that *Tmem108* also is related to BD. Furthermore, RNA sequencing showed that *Tmem108* expression is much higher in newly formed OLs than in other cells in the central nervous system (CNS) [17]. Therefore, these studies indicate that *Tmem108* may play a role in OL development and myelination.

In this study, *Tmem108* expression in the CC was higher in young mice than in adult mice and colocalized with OLs in young mice CC, implying promising function in myelination with the development. Intriguingly, myelin basic protein (MBP) was highly expressed in *Tmem108* mutant mice in immunohistochemistry (IHC) staining and western blots (WB) assay, and electron microscopes revealed hypermyelination in the CC of the mutant mice, especially early-onset myelination in small axons. Consistently, the cytological experiment showed that *Tmem108* inhibited OL progenitor cell (OPC) proliferation and mitigated the maturation of CC OLs by preventing the myelination of small-diameter axons. Moreover, *Tmem108* mutant mice exhibited manic behavior after acute restraint stress and were susceptible to drug-induced epilepsy. This study disclosed the function of *Tmem108* in CC, which may also provide a new role of *Tmem108* involving BD pathogenesis via regulating the myelination.

## Materials and methods

### Animals

*Tmem108* mutant (*Tmem108*-*LacZ*; MMRRC: 032633-UCD) mice were described previously [16, 18, 19]. In brief, the first coding exon of *Tmem108* was replaced with the β-galactosidase/neomycin cassette. *Tmem108* mutant mice in the paper were *Tmem108*-*LacZ* homozygous (*Tmem108* –/–). Mice were fed in a room 12-h light/dark cycle, at 22–25℃, with ad libitum access to rodent chow diet and clean water. The experimental protocols were performed according to the "guidelines for the care and use of experimental animals" issued by Nanchang University for research about vertebrate animals. For in vivo experiment, surgery was performed with sodium pentobarbital anesthesia (50 mg/kg, intraperitoneal injection), and efforts were executed to minimize suffering and reduce the animal number. After the terminal experiments, mice were euthanized by carbon dioxide inhalation.

### Reagents

X-gal (5-Bromo-4-chloro-3-indolyl-β-d-galactopyranoside) was purchased from Sigma-Aldrich (B4252, 30 mg/ml for staining); Pilocarpine hydrochloride and scopolamine methyl-bromide were also purchased from Sigma-Aldrich; Other chemicals were purchased from Sangon Biotech (BBI Life Sciences CO. China).

Antibodies information as follows: Rabbit anti-β-Actin antibody (Santa Cruz Biotechnology, sc-1616-R; 1:2000 for blotting); Rat anti-MBP antibody (Millipore, MAB386; 1:2000 for blotting; 1:1000 for staining); Rabbit anti-TMEM108 antibody (1:1000 for blotting) was kindly presented by Dr. J. Liu [20]; Goat anti-rabbit IgG poly-HRP secondary antibody (32260) and Goat anti-rat IgG poly-HRP secondary antibody (31471) were purchased from Thermo Fisher Scientific (1:2000 for blotting); Mouse anti-Ki67 (BD Biosciences, 550609; 1:1000 for staining); Rabbit anti-Olig2 antibody (Millipore, AB9610, 1:1000 for staining); Rabbit anti-PDGFRα antibody (Cell Signaling Technology; 3174; 1:500 for staining); Rat anti-PDGFRα antibody (BD Biosciences; 558774; 1:200 for staining); Mouse anti-APC antibody (CC1, Millipore Sigma, MABC200, 1:800 for staining); Rabbit anti-Caspase3 antibody (Cell Signaling Tech, 9662; 1:1000 for staining); Alexa Fluor 488/568 goat anti-rabbit lgG (Thermo Fisher Scientific, A32731, A11011; 1:1000 for staining), Alexa Fluor 488/568 goat anti-mouse lgG (Thermo Fisher Scientific, A-11029, A-11031; 1:1000 for staining).

### Behavioral analysis

For the forced swimming test (FST), mice were forced to swim in a two-liter beaker filled with about fifteen-centimeter-height water for 6 min. A camera monitored mice movements with tracking software (Video freeze version 2.5.5.0, Med Associate Inc.). The immobility in the last four min was obtained for statistical analysis.

According to the previous study, the pilocarpine model was conducted [21, 22]. In order to minimize the peripheral side effects, mice were injected with scopolamine methyl-bromide (2 mg/kg mice weight, intraperitoneal injection) 30 min before pilocarpine hydrochloride (dissolved in 0.9% saline, 200 mg/kg mice weight, intraperitoneal injection) treatment. Then, mice were injected with pilocarpine (100 mg/kg mice weight) every 30 min. Behavioral seizure score was according to the criteria by Racine [21]: stage 0, no seizure; stage 1, head nodding; stage 2, sporadic full-body shaking and spasms; stage 3, chronic full-body spasms; stage 4, jumping, shrieking, and falling over; stage 5, violent convulsions, falling over and dying.

### Western blots (WB)

WB was conducted as our previous research, and white matter (CC and medulla) and gray matter (top layers of cortex) separation were according to the previous study [23]. We homogenized the tissues in lysis buffer (0.1% SDS, 0.5% sodium deoxycholate, 1% NP-40, 1 mM EDTA, 1 mM PMSF, 1 mg/ml aprotinin, leupeptin, and pepstatin A protease inhibitors, in 1 × DPBS). The protein was separated by SDS-PAGE and then transferred to a nitrocellulose membrane. After being blocked, the membrane was incubated with primary antibody and HRP-coupled secondary antibody in turn. In the last, the immunoreacted bands were captured by an enhanced chemiluminescence system (Bio RAD), and the band intensities were performed with ImageJ software.

### Immunohistochemistry (IHC) staining

The mice brain's coronal setions (30 µm) were prepared by microtome (Leica CM1950) for IHC. After incubated in a citrate buffer for antigen repair, sections were permeabilized with a 20% tween for 20 min. Next, the sections were blocked for 1 h at room temperature and then incubated with primary antibody at 4 °C overnight. Afterward, sections were exposed to the secondary antibody for 2 h in the dark at room temperature. Finally, the sections were transferred to the slides and mounted with coverslips. The images were captured with an inverted fluorescence microscope (Olympus FSX100). DAPI was used to identify the cellular nuclei.

### EdU labeling

According to the previous study, the EdU labeling was conducted with minor modified [24]. Daily intraperitoneal injection of 3 mg EdU (Carbosynth, NE08701) per gram body weight (P8 mice) was performed to label dividing cells for 1 week. 24 h after the last injection, brain sections were collected, rinsed 3 times in PBS (phosphate-buffered saline), and then permeabilized for 30 min in PBS with 0.5% Triton X-100. After another 3 washes in PBS, the brain sections were incubated in a freshly-made EdU development cocktail (100 mM Tris-buffered saline pH 7.4, 2 mM CuSO4 (Sangon Biotech A600063, CAS #7758-99-8), 2 μM 6-FAM Azide (Lumiprobe #35130) and 100 mM Sodium Ascorbate (Sigma-Aldrich A7631, CAS#134-03-2). Following DAPI staining for 10 min, the brain sections were washed 3 times in PBS and mounted for imaging.

### Quantitative real-time PCR (qPCR)

Total RNA was isolated from mice brains according to the manufacturer's instructions of TRIzol Reagent (Invitrogen), and complementary DNA (cDNA) was synthesized following the manufacturer's protocol of High-Capacity cDNA Reverse Transcription Kit (Thermo Fisher Scientific, 4368814). The qPCR primer sets as below: *Tmem108* (5′-CCTGAGCTACTGGAACAATGCC-3′ and 5′-CAGTGTCTCGATAGTCGCCAT TG-3′), and *Gapdh* (5′-CATCACTGCCACCCAGAAGACTG-3′ and 5′-ATGCCAGTGAGCTTCCCGTTCAG-3′). qPCR was carried out by the StepOnePlus Real‐Time PCR system (Applied Biosystems) using the mix. qPCR was performed as described previously [25]. mRNA expression levels were normalized to the reference gene Gapdh using a ΔCT method.

### X-gal staining

X-gal is an inert chromogenic substrate for β-gal, and β-gal hydrolyzes X-gal into colorless galactose and 4-chloro-3-brom-indigo, forming an intense blue precipitate. X-gal staining was carried out as our previous study [16, 26]. In brief, the coronal sections of *Tmem108* mutant mice were prepared by microtome (Leica CM1950) and permeabilized with a detergent solution (0.01% sodium deoxycholate, 0.02% NP-40, 2 mM MgCl2 in 0.1 M pH 7.4 phosphate buffer) at 4 °C. After incubated in staining solution (5 mM potassium ferricyanide, 5 mM potassium ferrocyanide, 2 mg/ml X-gal in detergent solution) overnight at 37 °C, the sections were transferred to the slides and mounted with coverslips. Finally, the images were captured with an inverted fluorescence microscope (Olympus FSX100).

### Electron microscopy

For electron microscopy, young (P14) and adult (P60) male mice were perfused through the heart with phosphate buffer (PB 0.1 M, pH 7.4) followed by 2% paraformaldehyde with 0.5% glutaraldehyde in PB. After carefully dissecting the brain, the CC was immediately put into 2.5% glutaraldehyde incubating overnight at 4 °C and rinsed in PB 5 min triple times. Then at room temperature, the CC was postfixed in1% osmium tetroxide for 1 h, dehydrated through 30% and 50% ethanol 15 min in turn, and immersed in 70% uranyl acetate saturating ethanol overnight. Afterward, the sample was dehydrated through 80% and 95% ethanol 15 min, in turn, followed by incubation of 100% ethanol 40 min twice. The dehydrated sample was infiltrated in epoxypropane (30 min), epoxypropane:ethoxyline resin (1:1, 2 h), epoxypropane:ethoxyline resin (1:2, 1 h), and ethoxyline resin (Ephon812, 2 h) in a gelatin capsule in turn. Then, the sample was polymerized in an oven at 45 °C 12 h and 65 °C 48 h. Next, ultra-thin Sects. (70 nm) of the transversal cut CC axons were prepared on an ultramicrotome (LKB-Nova, Sweden). The sections were transferred onto 100 mesh copper grids followed rinsed with ddH_2_O 15 min triple times and stained in lead citrate 15 min followed with ddH_2_O 10 min triple times. Finally, the sections on the grids were examined on a transmission electron microscope (JEOL, JEM-2100, Japan). The *g*-ratio of a myelinated axon was calculated as the axonal diameter and fiber diameter ratio.

### Statistical analysis

Values of all data are mean ± SEM (standard error of the mean). Statistical analysis was carried out by GraphPad Prism 6.01. The statistical significance between the mutant and control mice was calculated by two‐way ANOVA (analysis of variance) and a two-tailed student t-test. The difference was defined as significant if the p-value < 0.05.

## Results

### Higher *Tmem108* expression of CC OLs in young mice than in adult mice

**Fig. 1:**
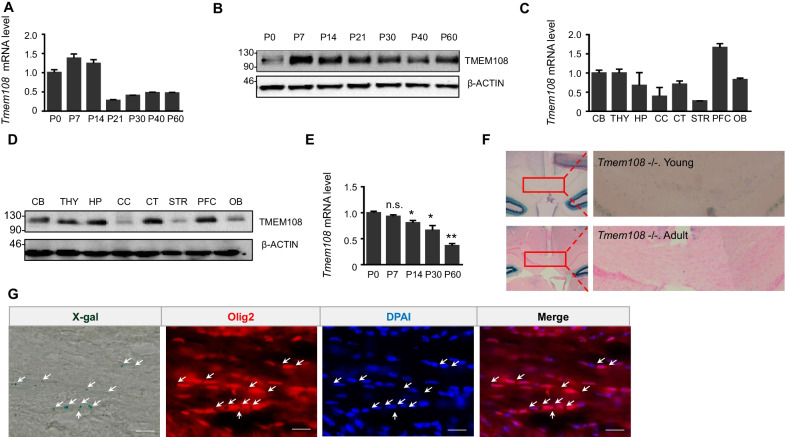
*Tmem108* expression profile in several areas of the brain. **A** Relative expression of *Tmem108* in postnatal mice brain was quantified by qPCR. Gapdh was used as an internal control (Internal control of the below is same in qPCR assay), and *Tmem108* expression in P0 mice was defined as 1; Wild-type male mice per group, n = 5. **B** Representative image of TMEM108 level in postnatal mice brains verified by western blotting. β-ACTIN was used as an internal control (Internal control of the below is the same in western blotting). **C** *Tmem108* relative expression in the different areas of the adult mice brain. *Tmem108* expression in CB was defined as 1; Wild-type male mice, n = 3; *CB* cerebellum, *THY* thalamus, *HP* hippocampus, *CC* corpus callosum, *CT* cerebral cortex, *STR* striatum, *PFC* prefrontal cortex, *OB* olfactory bulb. **D** Representative image of TMEM108 level in different areas of adult mice brain verified by western blotting. **E** *Tmem108* relative expression of CC decreased in postnatal mice development. *Tmem108* expression in P0 mice CC was defined as 1; Wild-type male mice per group, n = 3 (Unpaired T-test were made to compare with P0 data, n.s., not significant, * p < 0.05, ** p < 0.01). **F** *Tmem108* expression in young mice CC was higher than in adult mice CC by X-gal staining. *Tmem108* −/− mice per group, n = 3. **G** Co-staining indicated *Tmem108* expression mostly colocalized with OLs in the corpus callosum of young mice. X-gal staining represented β-gal expression downstream of the *Tmem108* promoter, and the arrows showed the X-gal staining dots in *Tmem108* mutant mice (P14, *Tmem108* −/− mice n = 3, scale bar = 20 μm)

*Tmem108* expression was verified by qPCR and WB (Fig. 1). For the whole brain of wild-type mice, qPCR results showed that *Tmem108* had a high expression in the young mice (Fig. 1A), and the protein level of *Tmem108* was similar to the mRNA level (Fig. 1B). qPCR and WB were utilized to check *Tmem108* expression in different brain areas (Fig. 1C, D), including cerebellum (CB), thalamus (THY), hippocampus (HP), corpus callosum (CC), cerebral cortex (CT), striatum (STR), prefrontal cortex (PFC) and olfactory bulb (OB). Though CC and STR seemed to like having a low expression of *Tmem108*, the results supported *Tmem108* expression in both areas.

The highest expression cell type in the mice brain was newly formed OLs (Additional file 1: Fig. S1) [13]. CC was considered an area with high myelinated axons, and therefore, CC was recruited to explore the role of *Tmem108* in the OL lineage cells. Focusing on CC, *Tmem108* was mainly expressed in PDGFRα^+^ Olig2^+^ cells (OPCs) in P7 mice (Additional file 1: Fig. S2A-B), and its expression profiles changed in P14 mice (Additional file 1: Fig. S2C-D), without difference among OPCs (PDGFRα^+^), OLs (CC1^+^) and the double negative (PDGFRα^−^ CC1^−^) cells. Considering *Tmem108* being highly expressed in newly formed OLs (Additional file 1: Fig. S1), the double negative cells in the CC may represent premyelinating OLs.

*Tmem108* expression decreased in postnatal development by qPCR assay, and *Tmem108* mRNA relative level in P7 mice CC was about two times that in P60 mice CC (Fig. 1E). Meanwhile, X-gal staining confirmed that *Tmem108* expression was higher in young mice CC than in adult mice CC (Fig. 1F). Co-staining assay suggested that X-gal was remarkably co-stained with OL marker Olig2 of CC in the young mice (Fig. 1G).

### Hypermyelination of the CC in *Tmem108* mutant mice

Myelin sheath can be observed by transmission electron microscopy, presenting thick, dark closed curves around myelinated axons. In this research, the myelinated axons of the CC from young and adult perfused male mice were examined under an electron microscope. The myelinated axons' ultrastructure was obtained for statistics, and the percentage of the myelinated axons in the total axons was reported. Littermate male mice were used to minimize the background effect from other genes between the mutant mice and the control mice.

**Fig. 2:**
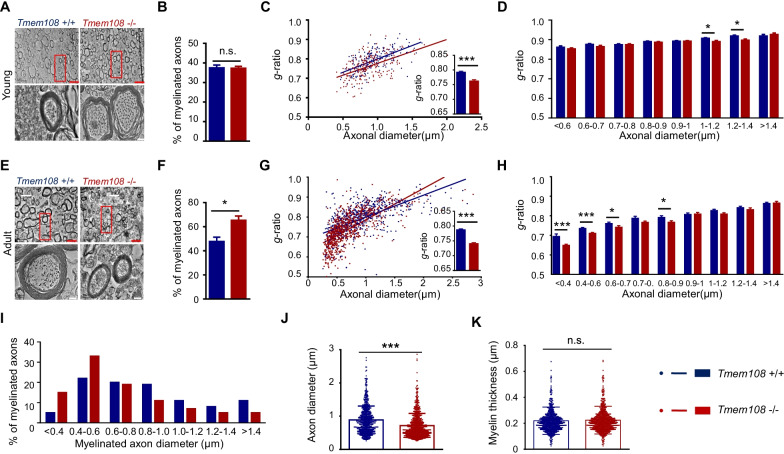
Hypermyelination of the corpus callosum in *Tmem108* mutant mice. **A** Representative electron microscopy of axons in the CC of young animals at P14. Same littermate male mice were used. **B** Percentages of CC myelinated axons were quantified in the young mice(P14). **C** Scatter plot of *g*-ratio values and *g*-ratio mean in the CC of the young mice(P14). **D** Mean *g*-ratio values of CC myelinated axons with different diameters in the young mice(P14). **E** Representative electron microscopy of axons in the CC of adult mice (2 M). Same littermate male mice were used. **F** Percentages of CC myelinated axons were quantified in the adult mice. **G** Scatter plot of *g*-ratio values and *g*-ratio mean in the CC of the adult mice. **H** Mean *g*-ratio values of CC myelinated axons with different diameters in the adult mice. (Red scale bar = 2 μm, white scale bar = 200 nm; Male mice per group n = 3, over 200 fibers per each type of animal were analyzed; Values were means ± SEM; Unpaired T-test were made between the groups; n.s., not significant, * p < 0.05, *** p < 0.001). **I**–**K** Small-diameter CC axons in *Tmem108* mutant mice were readily myelinated. **I** Percentage of myelinated axons with different diameters in the total myelinated axons. **J** Diameter of myelinated axons in the adult mice. **K** Myelin thickness of CC axons in the adult mice (*Tmem108* + / + mice n = 3, axon fiber n = 684; *Tmem108* −/− mice n = 3, axon fiber n = 757; Unpaired T-test were made between the groups; n.s., not significant)

Because myelination increases with mice's development, it was not surprising that the percentage of the myelinated axons in the adult mice was 10–30% higher than in the young mice (Fig. 2B, F). The *g*-ratio value of a myelinated axon is defined as the ratio of the axonal diameter and myelinated fiber diameter, considered a typical myelination indicator. Accordingly, a high value of *g*-ratio indicates a low value of relative myelin thickness. Myelinated axon percent in *Tmem108* young mutant mice CC resembled the control mice CC (Fig. 2B), but the *g*-ratio value of CC in the young mutant mice was lower than the control mice (Fig. 2C), implying hypermyelination in the mutant mice. In line with expectation, hypermyelination of the CC in the adult mutant mice was observed (Fig. 2G), and myelinated axon percent in the adult mutant mice was higher than the control mice (Fig. 2F). Furthermore, the mean *g*-ratio values of the CC axons with different diameters in the mice were counted and statistically analyzed (Fig. 2D, H). Notably, thin fibers in the myelinated axons of adult mice CC processed a small value of *g*-ratio (Fig. 2H).

### Preferred myelination of thin axons in *Tmem108* mutant mice

Electron microscopy also demonstrated the enhancement of OL maturation in the mutant mice. In the adult mice, myelin sheath was not different between the mutant and the control mice (Fig. 2E, K), though their *g*-ratio was distinct (Fig. 2G). The percentage of diverse diameter fibers in total myelinated axons was investigated (Fig. 2I). Strikingly, nearly half myelinated axons in the mutant mice were thin fibers, with the axon diameter no more than 600 nm (Fig. 2I), and the diameter of myelinated OLs in the mutant mice was smaller than that in the control mice (Fig. 2J).

### High expression of *Mbp* in *Tmem108* mutant mice

**Fig. 3:**
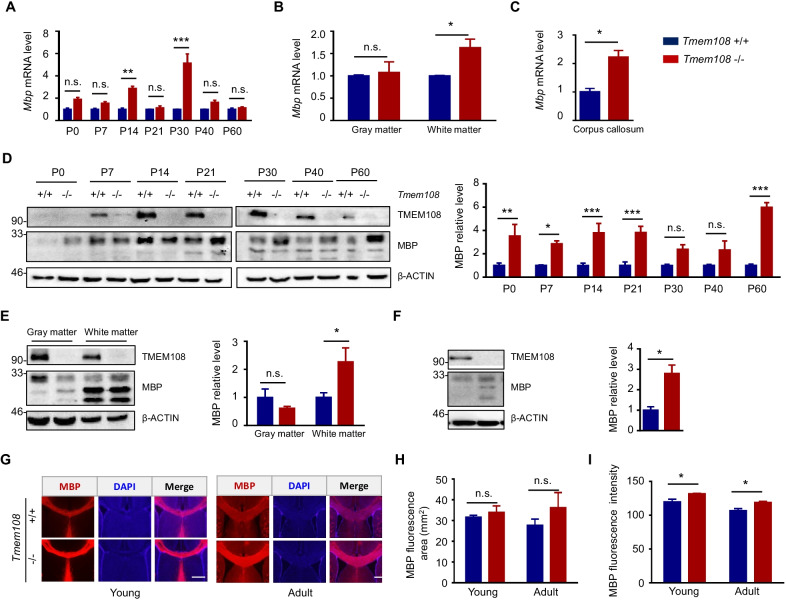
High expression of *Mbp* in *Tmem108* mutant mice. **A** Quantified relative mRNA expression of *Mbp* by qPCR in the whole brain (Male mice per group n = 3; Two-way ANOVA analysis, postnatal day P-value < 0.001, genotype P-value < 0.01, interaction P-value < 0.001, and Sidak's multiple comparisons test was conducted on indicated postnatal day). **B**, **C** Relative mRNA expression of *Mbp* by qPCR in gray matter, white matter (**B**) or corpus callosum (**C**) (Adult male mice, *Tmem108* + / + mice n = 3, *Tmem108* −/− mice n = 3; Unpaired T-test were made between the groups). **D** Representative image of MBP level in postnatal mice brain by western blotting (left panel); Quantification of MBP level in the western blotting (*Tmem108* + / + mice n = 3, *Tmem108* −/− mice n = 3; Two-way ANOVA analysis, postnatal day P-value = 0.0441, genotype P-value = 0.0122, interaction P-value = 0.0024, and Sidak’s multiple comparisons test was conducted at each postnatal day). **E–F** Representative image of MBP level in different areas of adult mice brain by western blotting (left panel); Quantification of MBP level in the western blotting (right panel), including gray matter, white matter (**E**), and CC (**F**) (T-test analysis, *Tmem108* + / + mice n = 5, *Tmem108* −/− mice n = 5; Two-way ANOVA and unpaired T-test analysis were used). **G**–**I** High MBP fluorescence intensity in the corpus callosum of *Tmem108* mutant mice. **G** Representative images of MBP staining in mice CC. **H** MBP fluorescence area of CC in *Tmem108* mutant mice (*Tmem108* −/−) was not different from the control mice (*Tmem108* + / +). **I** MBP fluorescence intensity of CC in *Tmem108* mutant mice was higher than the control mice (Scale bar = 200 μm; Male mice per group, n = 3, unpaired T-test analysis). (Values are means ± SEM; n.s., not significant, *p < 0.05, **p < 0.01, **p < 0.001)

*Mbp* mRNA levels and MBP protein were examined in the postnatal mice brain (Fig. 3A, D), in gray matter and white matter of adult mice (Fig. 3B, E), and CC of adult mice (Fig. 3C, F). *Mbp* mRNA level seemed consistent with MBP protein level in the mutant mice (Fig. 3A–F). Whole-brain MBP level in postnatal mice was quantified by WB (Fig. 3D), and MBP expression was higher in *Tmem108* mutant mice than in the control mice after birth. According to the previous study [23], gray matter and white matter were separated, and the latter were considered brain areas with plenty of myelinated axons. WB indicated that white matter in the mutant mice brain had more MBP protein than in the control brain (Fig. 3E). Consistent with anticipation, MBP in CC of the mutant mice brain was higher than the control brain (Fig. 3F).

Although *Tmem108* mutant did not alter the CC area (Fig. 3G, H), cerebral cortex structure, and the hippocampus construction (Additional file 1: Fig. S3), MBP fluorescence intensity of the brain CC in *Tmem108* mutant mice elevated (Fig. 3G, I).

### The proliferation of OL increased in the CC of the young mutant mice

**Fig. 4:**
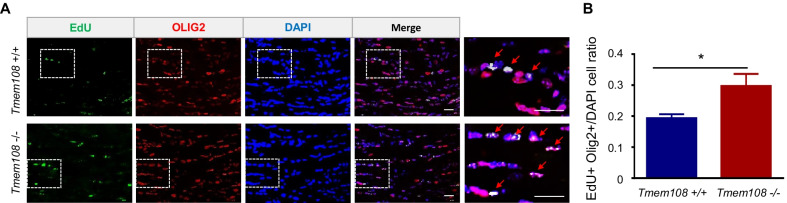
The proliferation of OL increased in the CC of the young mutant mice. **A** Representative CC images in young mice at P15 co-staining EdU with Olig2, EdU injection once a day from P8 to P14. The last-right panel was signified the white rectangular area in the Merge panel. **B** Quantified double-positive (EdU^+^ Olig2^+^) ratio in the young mice CC. (Scale bar = 20 μm; Mice per group n = 3; Values are means ± SEM; Unpaired T-test were made between the groups; *p < 0.05)

Myelination begins relatively late in the development of mice until after birth, and myelin sheaths are first seen at P11. Increased myelination occurs during neonatal development of the mice [27]. We speculated that young mice before P11 might have a high proliferation of OPCs or newly formed OLs. EdU was injected into P8 mice once a day for 7 days. CC of the P15 mice was co-stained in EdU with Olig2 (Fig. 4A). EdU^+^ Olig2^+^ cell density increased in the mutant mice (Fig. 4B), which indicated high proliferation of the OPCs or newly formed OLs in the mutant mice. Moreover, in other words, the result suggested that *Tmem108* inhibited OPC and newly formed OLs proliferation.

### The maturation of CC OLs in *Tmem108* mutant mice was enhanced

**Fig. 5:**
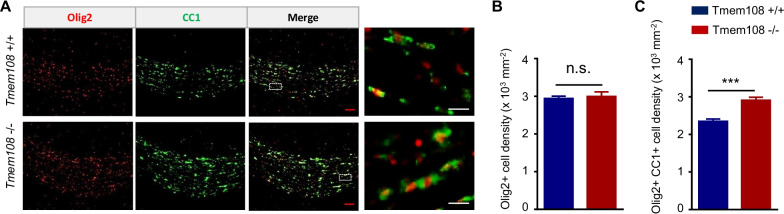
Maturation of CC oligodendrocytes in *Tmem108* mutant mice increased. **A** Representative images of CC in the adult mice co-staining CC1 with Olig2. The dotted areas in the left panel were enlarged and shown on the right panel. **B** Quantified Olig2 positive cell density of CC in the adult mice. **C** Quantified double-positive (Olig2 + and CC1 +) cell density of CC in the adult mice. (Red scale bar = 50 μm, white scale bar = 10 μm; Male mice per group n = 5; Values are means ± SEM; Unpaired T-test were made between the groups; n.s., not significant, *** p < 0.001)

**Fig. 6:**
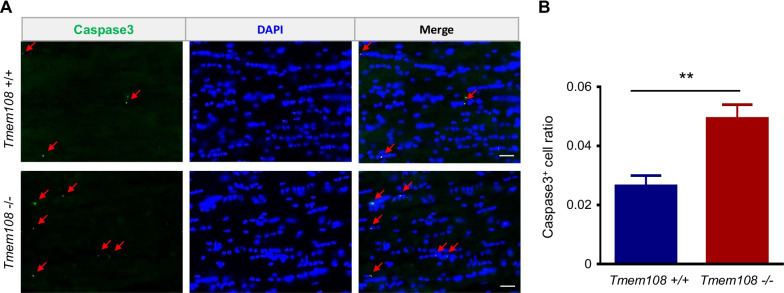
High-level apoptosis of CC cells in *Tmem108* mutant mice. **A** Representative images of CC in the adult mice staining apoptosis marker (Caspase3). The arrows showed the positive dot of Caspase3 staining. **B** Quantified Caspase3 positive cell density of CC in the adult mice. (Scale bar = 20 μm; Mice per group n = 6; Values are means ± SEM; Unpaired T-test were made between the groups; **p < 0.01)

To investigate the OLs maturation in the *Tmem108* mutant mice, we utilized CC1 staining to evaluate the maturation of CC OL lineage cells in adult mice (Fig. 5). OL lineage cells proliferation increased in the mutant mice, but OL lineage cells density did not change in adult mice (Fig. 5B). Therefore, Caspase3, as an apoptosis marker, was engaged in assessing the apoptosis condition in the *Tmem108* mutant CC area. The mutant mice displayed hyperapoptosis conditions in the CC area (Fig. 6). Intriguingly, mature OL lineage cells represented by Olig2 and CC1 double-positive cells increased in the mutant mice (Fig. 5C), implying that *Tmem108* mitigated the maturation of CC OLs.

### Mania-like behavior and easily induced epilepsy in *Tmem108* mutant mice

**Fig. 7:**
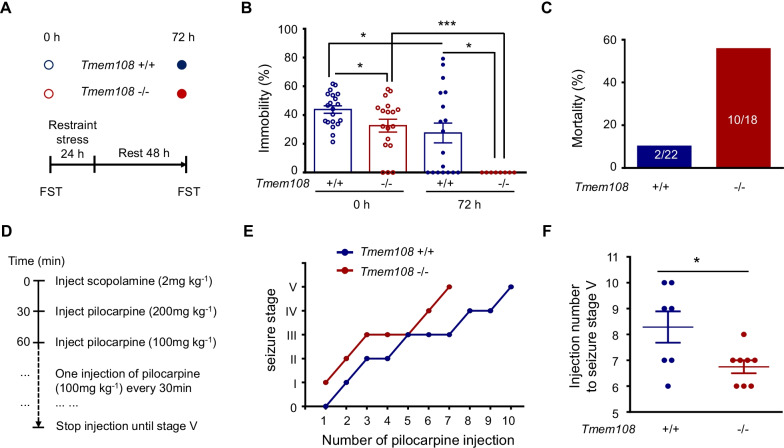
*Tmem108* mutant mice exhibited mania-like behavior and were easily induced epilepsy. **A** Schematic of forced swimming test (FST) with restraint stress; FST were recorded before restraint stress and two-day interval after restraint stress. **B** Comparing the immobility with the control mice before and after restrain stress, *Tmem108* mutant (*Tmem108* −/−) mice exhibited mania-like behavior in the tests (*Tmem108* + / + mice n = 22, *Tmem108* −/− mice n = 18). **C** *Tmem108* mutant mice had serious mortality in the restraint stress. **D** Schematic of seizure inducement. Scopolamine was injected 30 min before applying pilocarpine to minimize the peripheral side effects. **E** Representative time courses of seizure development by repeated pilocarpine injection. Mice of two genotypes were subjected to pilocarpine injection every 30 min and scored for the seizure stage. **F** An increased number of pilocarpine injections were needed to reach stage 5 seizure for *Tmem108* mutant mice (*Tmem108* + / + mice n = 7, *Tmem108* −/− mice n = 8). (Unpaired T-test analysis; * p < 0.05, *** p < 0.001)

*Tmem108* mutant mice display anti-depression behavior (mania-like) behavior in the previous study [16]. In this study, 24-h restraint stress worsened the mania behavior in FST (Fig. 7). Over half of *Tmem108* mutant mice exhibited severe mania-like behavior during the restraint stress and died of physical exertion (Fig. 7C). Furthermore, after the restraint stress, the remaining mutant mice struggled desperately without rest in FST (Fig. 7B), indicating severe mania-like behavior in the mutant mice. Meanwhile, the control mice could be separated into the mania-like group, depressive group, and resistant group (Fig. 7B).

Due to the similarities and the behavioral manifestation between pathophysiological mechanisms and the chronic seizures for their spontaneous and recurrent characteristics, the pilocarpine injection model is considered a classic experimental protocol to mimick human temporal lobe epilepsy [28]. The pilocarpine model was utilized to evaluate the potential epilepsy of the mutant mice. In order to avoid the peripheral side effects, scopolamine block was injected before the pilocarpine treatment (Fig. 7D). The mice were observed continuously for behavioral seizures after each pilocarpine injection. *Tmem108* mutant mice quickly reached seizure stage 5 compared with the control mice (Fig. 7E, F), indicating *Tmem108* involved the occurrence of induced epilepsy.

## Discussion

### Abnormal myelin development and the mental diseases

Although SCZ and BD account for 2–4% of the world population, the pathogenesis and treatment of SCZ and BD are unclear and unsatisfied [29, 30]. Strikingly, imaging and autopsy studies not only show that abnormal white matter is a common neurobiological change in BD and SCZ patients [4] but also reveal that SCZ patients are accompanied with dysregulation of OL related processes, such as myelination developmental disorder, abnormal expression of myelination gene and number changes of OLs [31, 32]. However, the molecule linking abnormal myelination with mental diseases is unclear.

The myelin sheath is composed of bilayer lipids as the frame, with proteins embedded as one of the plasma membranes. Most of the proteins in the myelin sheath are transmembrane proteins, such as MBP and proteolipid protein. In these proteins, MBP accounts for 30% of the total myelin protein in the CNS and is critical for myelination [33, 34]. MBP expression was enhanced in *Tmem108* mutant mice via WB and IHC staining, and the mutant mice also exhibited hypermyelination by electron microscopy.

The myelin sheath in mature OL acts as an external insulator for current conduction, facilitating rapid saltatory impulse conduction with reduced axonal diameters. Moreover, myelin also provides essential nutritional support for myelinated neurons. Myelinated fibers are widely distributed in the brain, and myelin sheath is essential for maintaining neural circuits. Accordingly, hypomyelination or hypermyelination of OL deriving from abnormal myelination may be one of the bases of cognitive impairment in SCZ and BD, also relating to poor prognosis [31, 35].

The cause of abnormal behaviors in *Tmem108* mutant mice is complicated. *Tmem108* mutant mice in this research were *Tmem108* null mice. In the previous studies, *Tmem108* knockout leads to a decrease of adult neurogenesis in the dentate gyrus [16] and impairs spine development and glutamate transmission [18]. So, neuronal deficits in the mutant mice may also induce manic behavior and easily be induced epilepsy. Therefore, single or both neuronal and oligodendrocyte loss of TMEM108 in the brain may contribute to abnormal behaviors. Condition knockout *Tmem108* in OLs or neurons will provide direct evidence for the behavior change.

### Potential multiple functions of *Tmem108* in the CNS

*Tmem108*, also known as Retrolinkin [20, 36, 37], is located on human chromosome 3q21. GWAS found that TMEM108 is not only related to substance addiction [38], smoking withdrawal [39], and alcohol addiction [40–43], but also is a susceptibility gene of SCZ [12, 13, 15] and BD [12–14].

O'Donovan et al*.* found that the SNP (rs7624858) mutation in the intron of *Tmem108* is related to SCZ [15, 44]and speculated that the site caused *Tmem108* to become a susceptibility gene of SCZ by affecting gene expression. Jiao et al*.* disclosed that *Tmem108* mutant mice are impaired in spatial memory, and fear startles contextual memory and is more sensitive in PPI performance [18], a classic and plausible psychophysiological measurement of sensorimotor gating for SCZ in rodents and humans [45, 46].

The nature of the severe mental illness has been debated for more than one century. According to the prevailing manuals, International Classification of Diseases, BD, and schizophrenia reveal striking similarities, and the difference is that sensory gating and cognitive impairments are less pronounced in BD patients [47]. BD and schizophrenia consistently ranked among the leading causes of disability worldwide [48, 49], with similarities across multiple levels, such as overlapping brain structural [50, 51] and shared genetic risk factors [52–55]. BD and schizophrenia are severe psychiatric disorders with high heritability, but to date, unknown etiology, sharing genetic risk factors, and a possible illness mechanism is abnormal myelination [11, 56, 57].

Although *Tmem108* mutant impairs adult neurogenesis of the mice, it does not induce depression-like behavior but stirs manic-like behavior, suggesting *Tmem108* is higher correlating with BD than depression [16]. Strikingly, one recent GWAS screened BD risk loci in the Han Chinese population, covering 1822 BD patients and 4650 control individuals, and the data was replicated analysis. After finally multiple analyses between Han Chinese and European populations, a new SNP (rs9863544) in BD patients were found, locating in the upstream regulatory region of the *Tmem108* gene [14]. *Tmem108* expression change may be one of the onset reasons of the related psychiatry diseases.

Researchers have debated whether severe, chronic irritability without episodic mania constitutes a developmental phenotype of BD [5]. Neurobiological models of BD emphasize white matter aberrant development, and white matter microstructure is often described as fractional anisotropy, which is positively associated with the smaller axon diameter and increased axon packing density [5].

### The potential molecular mechanism of *Tmem108* regulating myelination

In 2014, Zhang et al*.* purified eight representative cell populations from the cortex and generated the RNA transcriptome database for the different types of cells [17] (Additional file 1: Fig. S1), which indicates *Tmem108* is expressed much higher in newly formed OLs than in other OL lineage cells or neurons. Intriguingly in previous research, *Tmem108* was found highly expressed in the granular cells of the dentate gyrus [16, 18]. In this research, OL lineage cells exhibited higher *Tmem108* expression than other cells in mice CC. *Tmem108* mutant mice had manic-like behavior and were more active than the control group in forced swimming and tail suspension experiments [16]. Moreover, 24-h restraint exacerbated the manic-like behavior of the mutant mice, and the mutant mice were easily induced epilepsy by pilocarpine, which may be partially related to the abnormal myelination.

Although *Tmem108* expression was low in the CC of adult mice without X-gal staining detection, its expression was detectable in young wild-type mice and could be colocalized with Olig2 positive cells by utilizing the gene reporter mice. *Tmem108* was highly expressed in the cerebral cortex and hippocampus, with low expression in the CC; no alteration was found in the CC area, the cerebral cortex and the hippocampus construction in *Tmem108* mutant mice. However, MBP staining suggested no difference between *Tmem108* mutant mice and the control mice (Additional file 1: Fig. S4), which was inconsistent with high MBP in the mutant mice CC. The main reason may be the lower density of the OLs in the cerebral cortex than CC.

To explore how *Tmem108* inhibited proliferation and myelination of OL cells, gene expression with myelination regulation [58] was detected by qPCR. Intriguingly, *Tcf4* was also expressed highly besides myelin regulatory factor (*Myrf*) in all three brain areas of *Tmem108* mutant mice (Additional file 1: Fig. S5). In previous research, *Tmem108* was reported to involve adult neurogenesis by the Wnt signaling pathway. In CC, most genes with significantly altering expression were downstream of the Wnt signaling pathway, such as ID2, ID4 and TCF4/TCF7L2.

Wnt signaling plays a complicated role in the OL myelination, depending on the final effector in the signaling pathway. Canonical Wnt/β-Catenin signaling pathway strongly inhibits differentiation [59–61]. Under Wnt3a treatment, differentiation of OPC is strongly delayed or blocked [61], recruiting TCF4/TCF7L2 to β-Catenin target genes to promote proliferation [60, 62]. ID2 and ID4 are the potential targets of Wnt/β-Catenin/TCF4 signals in OL development.

Not surprisingly, β-Catenin decrease leads to enhancing the premyelinating OL. However, OL differentiation is not enhanced but reduced in *Tcf7l2* knockout mice [59, 62], and differentiation is delayed in β-Catenin inactivated mice [63], indicating the complexity of β-Catenin /TCF7L2. The potential mechanism is TCF7L2 interacting with HDAC1 (Histone deacetylases 1) and HDAC2, which repress the expression of differentiation inhibitors [59]. Thereby, TCF7L2 acts like a molecular switch, blocking or promoting OL differentiation by associating with the different binding partners [64]. We speculated that *Tmem108* regulated proliferation and myelination via the Wnt signaling pathway depending on different effectors.

The potential molecular mechanism of TMEM108 regulating myelination was complicated. Interaction between TMEM108 and Wnt signaling pathway increased its functional complexity and diversity. In OPCs, TMEM108 may inhibit proliferation via restricting β-Catenin/TCF7L2, and simultaneously, TMEM108 may also modulate differentiation via limiting HDAC/TCF4 interaction. In premyelinating OLs, TMEM108 may mitigate OL maturation through confining *Myrf* expression. The hypotheses need further research to verify them via in vivo and in vitro assays.

## Conclusion

This study disclosed that *Tmem108* inhibited OPC proliferation and mitigated the maturation of CC OLs, which may also provide a new role of *Tmem108* as a BD risk gene via regulating myelination.

## Supplementary Information

**Additional file 1: Fig. S1.** *Tmem108* expression profiles by cell type in the mice brain from RNA sequencing [13]. **Fig. S2** *Tmem108* expression in CC was mainly related to OPCs in P7 mice, and the expression profiles changed in P14 mice. A. Representative images of CC area of *Tmem108* mutant P7 mice. The arrows showed the X-gal staining dots, which indicated the potential areas of *Tmem108* expression. The right-below panel was signified the white rectangular area in the middle-below panel. B. Quantify of PDGFRα^+^X-gal^+^ cells percent in total X-gal^+^ cells in CC area (*Tmem108 −/−* mice, n = 4; Unpaired T-test analysis; * p < 0.05). C. Representative images of CC area of *Tmem108* mutant mice. The red arrow showed the X-gal staining dot relating to PDGFRα^+^ OL. The white arrows showed the X-gal staining dots associated with the CC1^+^ cell, and the yellow arrows indicated the X-gal staining dots connecting with double negative (PDGFRα^−^ CC1^−^) cells. The right-below panel was signified the white rectangular area in the middle-below panel. D. Quantify different types of X-gal^+^ cells percent in total X-gal positive cells in the CC area (*Tmem108 −/−* mice, n = 4; One-way ANOVA analysis; n.s., not significant). (Scale bar = 20 μm; Values are means ± SEM). **Fig. S3** No structural change of the cerebral cortex and the hippocampus in *Tmem108* mutant mice. A. No structural alteration of cerebral cortex in the mutant mice. B. No structural change of the hippocampus in the mutant mice. (Scale bar = 200 μm; Male adult mice per group n = 3). **Fig. S4** MBP expression in *Tmem108* mutant mouse cerebral cortex. A. Representative images of MBP staining in mice cerebral cortex. B-C. Quantified MBP fluorescence area (B) and fluorescence intensity of cerebral cortex in *Tmem108* mutant mice. There was no difference between the mutant mice and the control mice. (Scale bar = 200 μm; Male mice per group, n = 4, unpaired T-test analysis, n.s., not significant). **Fig. S5** Expression of myelination regulated genes in *Tmem108* mutant mice. A. Myelination regulated gene expression in the CC. B. Myelination regulated gene expression in the cerebral cortex. A. Myelination regulated gene expression in the striatum. D. Venn diagram of myelination regulated gene expression from CC, cerebral cortex, and striatum in *Tmem108* mutant mice. (Gapdh was used as an internal control, and gene expression in wild-type mice was defined as 1; Male adult mice per group n = 5; Unpaired T-test were made between the groups; * p < 0.05). **Table S1.** Sequences of qPCR primers.

## Data Availability

The datasets used or analyzed in our study are available from the corresponding author on reasonable request.

## Abbreviations

ANOVA: Analysis of variance
BD: Bipolar disorder
CB: Cerebellum
CC: Corpus callosum
CNS: Central nervous system
CT: Cerebral cortex
GWAS: Genome-wide association study
FST: Forced swimming test
HP: Hippocampus
IHC: Immunohistochemistry
MBP: Myelin basic protein
OB: Olfactory bulb
OL: Oligodendrocyte
OPC: OL progenitor cell
PFC: Prefrontal cortex
PPI: Pre-pulse inhibition test
qPCR: Quantitative real-time PCR
SEM: Standard error of the mean
SNP: Single nucleotide polymorphism
STR: Striatum
SCZ: Schizophrenia
*Tmem108*: *Transmembrane protein 108*
THY: Thalamus
WB: Western blots
X-gal: 5-Bromo-4-chloro-3-indolyl-β-d-galactopyranoside
