# Supplementary material for: Transmembrane protein 108 inhibits the proliferation and myelination of oligodendrocyte lineage cells in the corpus callosum
Source: Mol Brain. 2022 Apr 11;15:33. doi: 10.1186/s13041-022-00918-7 (PMC8996597; doi:10.1186/s13041-022-00918-7)

**Fig. S1** *Tmem108* expression profiles by cell type in the mice brain from RNA sequencing [13]


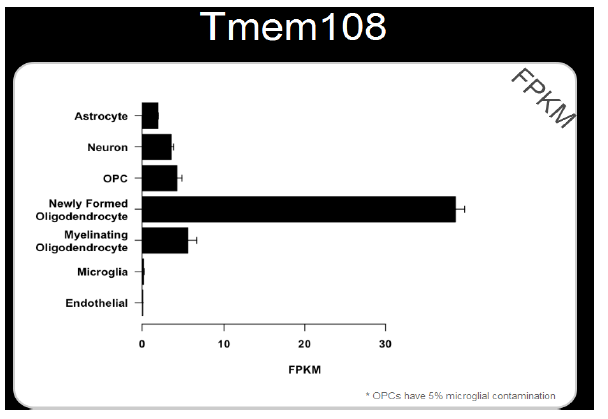


**Fig. S2** *Tmem108* expression in CC was mainly related to OPCs in P7 mice, and the expression profiles changed in P14 mice.

**A.** Representative images of CC area of *Tmem108* mutant P7 mice. The arrows showed the X-gal staining dots, which indicated the potential areas of *Tmem108* expression. The right-below panel was signified the white rectangular area in the middle-below panel. **B.** Quantify of PDGFRα^+^X-gal^+^ cells percent in total X-gal^+^ cells in CC area (*Tmem108 -/-* mice, n = 4; Unpaired T-test analysis; * p < 0.05). **C.** Representative images of CC area of *Tmem108* mutant mice. The red arrow showed the X-gal staining dot relating to PDGFRα^+^ OL. The white arrows showed the X-gal staining dots associated with the CC1^+^ cell, and the yellow arrows indicated the X-gal staining dots connecting with double negative (PDGFRα^-^ CC1^-^) cells. The right-below panel was signified the white rectangular area in the middle-below panel. **D.** Quantify different types of X-gal^+^ cells percent in total X-gal positive cells in the CC area (*Tmem108 -/-* mice, n = 4; One-way ANOVA analysis; n.s., not significant). (Scale bar = 20 μm; Values are means ± SEM)


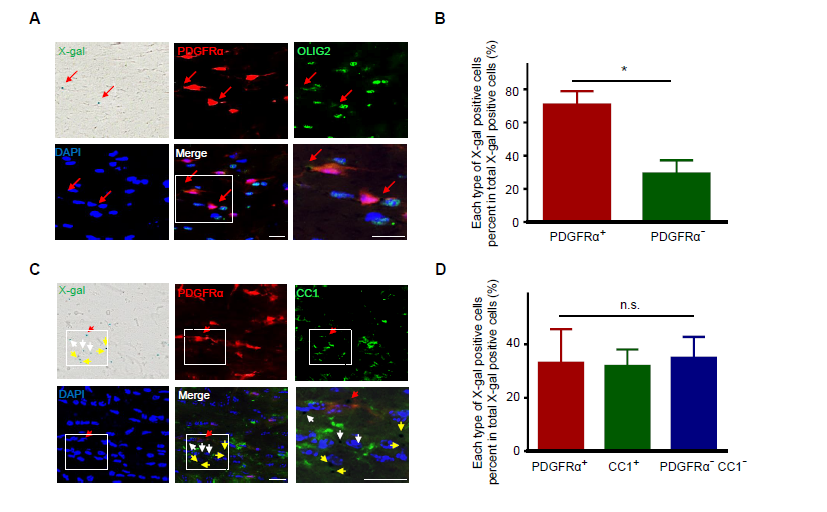


**Fig. S3** No structural change of the cerebral cortex and the hippocampus in *Tmem108* mutant mice

**A.** No structural alteration of cerebral cortex in the mutant mice. **B.** No structural change of the hippocampus in the mutant mice. (Scale bar = 200 μm; Male adult mice per group n = 3)


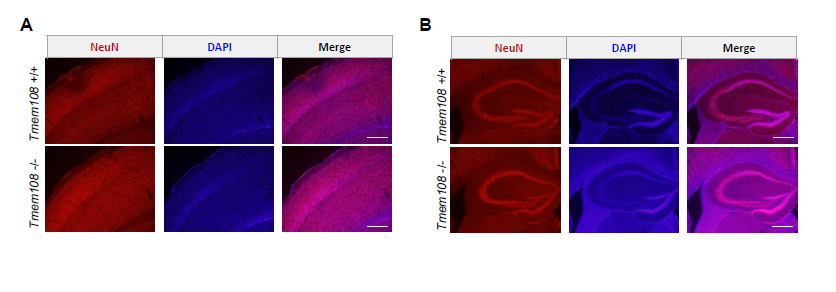


**Fig. S4** MBP expression in *Tmem108* mutant mouse cerebral cortex

**A.** Representative images of MBP staining in mice cerebral cortex. **B-C.** Quantified MBP fluorescence area (**B**) and fluorescence intensity of cerebral cortex in *Tmem108* mutant mice. There was no difference between the mutant mice and the control mice. (Scale bar = 200 μm; Male mice per group, n = 4, unpaired T-test analysis, n.s., not significant)


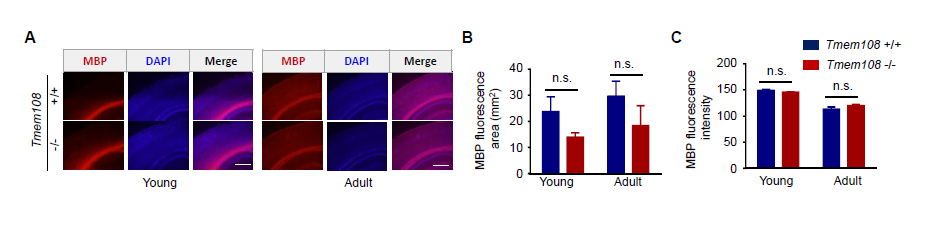


**Fig. S5** Expression of myelination regulated genes in *Tmem108* mutant mice

**A.** Myelination regulated gene expression in the CC. **B.** Myelination regulated gene expression in the cerebral cortex. **A.** Myelination regulated gene expression in the striatum. **D.** Venn diagram of myelination regulated gene expression from CC, cerebral cortex, and striatum in *Tmem108* mutant mice. (Gapdh was used as an internal control, and gene expression in wild-type mice was defined as 1; Male adult mice per group n = 5; Unpaired T-test were made between the groups; * p < 0.05)


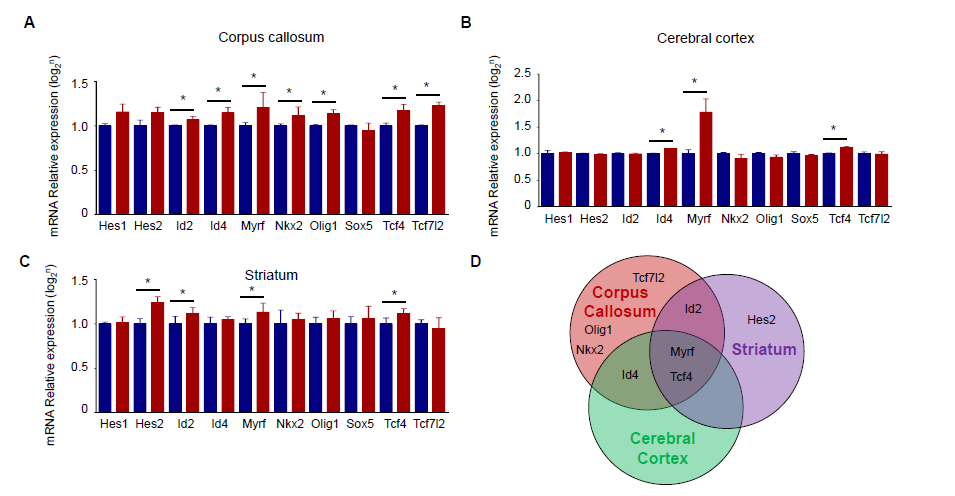

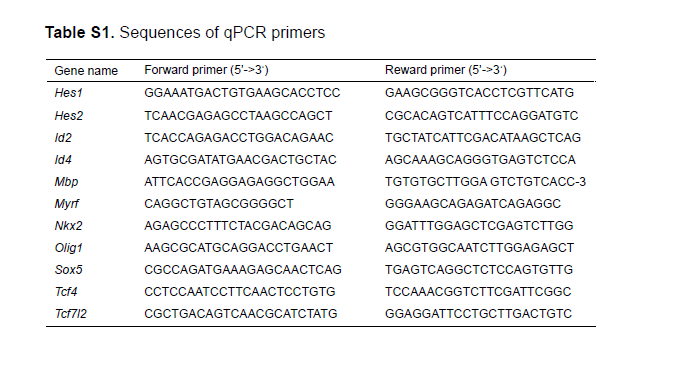

Supplement: Supplementary file 1 — Additional file 1: Fig. S1. Tmem108 expression profiles by cell type in the mice brain from RNA sequencing [13]. Fig. S2 Tmem108 expression in CC was mainly related to OPCs in P7 mice, and the expression profiles changed in P14 mice. A. Representative images of CC area of Tmem108 mutant P7 mice. The arrows showed the X-gal staining dots, which indicated the potential areas of Tmem108 expression. The right-below panel was signified the white rectangular area in the middle-below panel. B. Quantify of PDGFRα+X-gal+ cells percent in total X-gal+ cells in CC area (Tmem108 −/− mice, n = 4; Unpaired T-test analysis; * p < 0.05). C. Representative images of CC area of Tmem108 mutant mice. The red arrow showed the X-gal staining dot relating to PDGFRα+ OL. The white arrows showed the X-gal staining dots associated with the CC1+ cell, and the yellow arrows indicated the X-gal staining dots connecting with double negative (PDGFRα− CC1−) cells. The right-below panel was signified the white rectangular area in the middle-below panel. D. Quantify different types of X-gal+ cells percent in total X-gal positive cells in the CC area (Tmem108 −/− mice, n = 4; One-way ANOVA analysis; n.s., not significant). (Scale bar = 20 μm; Values are means ± SEM). Fig. S3 No structural change of the cerebral cortex and the hippocampus in Tmem108 mutant mice. A. No structural alteration of cerebral cortex in the mutant mice. B. No structural change of the hippocampus in the mutant mice. (Scale bar = 200 μm; Male adult mice per group n = 3). Fig. S4 MBP expression in Tmem108 mutant mouse cerebral cortex. A. Representative images of MBP staining in mice cerebral cortex. B-C. Quantified MBP fluorescence area (B) and fluorescence intensity of cerebral cortex in Tmem108 mutant mice. There was no difference between the mutant mice and the control mice. (Scale bar = 200 μm; Male mice per group, n = 4, unpaired T-test analysis, n.s., not significant). Fig. S5 Expression of myelination regulated genes in Tm [file 13041_2022_918_MOESM1_ESM.docx]
